# Appetite and its association with mortality in patients with advanced cancer – a Post-hoc Analysis from the Palliative D-study

**DOI:** 10.1186/s12904-023-01287-1

**Published:** 2023-10-26

**Authors:** Charlotte Goodrose-Flores, Stephanie E. Bonn, Caritha Klasson, Maria Helde Frankling, Ylva Trolle Lagerros, Linda Björkhem-Bergman

**Affiliations:** 1 Division of Neurobiology, Care Sciences and Society (NVS), Division of Clinical Geriatrics, Huddinge, Karolinska Institutet, Stockholm, Sweden; 2 Department of Medicine, Division of Clinical Epidemiology (KEP), Solna, Karolinska Institutet, Stockholm, Sweden; 3 Karolinska University Hospital, Thoracic Oncology Center, Theme Cancer, Solna, Stockholm, SE-171 64 Sweden; 4 Center of Obesity, Academic Specialist Center, Stockholm Health Services, Stockholm, Sweden; 5 Stockholms Sjukhem, Palliative Medicine, Mariebergsgatan 22, SE-122 19, Stockholm, Sweden

**Keywords:** Cancer, Palliative care, Appetite, Survival, Albumin, Fatigue

## Abstract

**Background:**

Loss of appetite is a common nutrition symptom in patients with cancer. Understanding the trajectory of appetite could be of clinical use for prognostication in palliative cancer care. Our primary aim was to explore the association between self-assessed appetite and mortality in patients suffering from advanced cancer. Secondary aims included the relation between fatigue, albumin levels and CRP/albumin ratio and mortality. We also aimed to study potential sex-differences in the associations.

**Methods:**

Post-hoc analyses were performed using data from the Palliative D-study comprising 530 patients with cancer admitted to palliative care. Appetite and fatigue were assessed with the Edmonton Symptom Assessment System (ESAS). Cox proportional hazards models were used to calculate Hazard ratios (HR) with 95% confidence intervals (CI) for exposures of appetite, fatigue, albumin and CRP/albumin ratio, and time from study inclusion to death or censoring. Analyses were also performed stratified by sex.

**Results:**

The follow-up time ranged between 7 to 1420 days. Moderate and poor appetite were significantly associated with a higher mortality rate compared to reporting a good appetite; HR 1.44 (95%CI: 1.16–1.79) and HR 1.78 (95%CI: 1.39–2.29), respectively. A higher mortality rate was also seen among participants reporting severe fatigue compared to those reporting no fatigue; HR 1.84 (95%CI:1.43–2.36). Participants with low albumin levels (< 25 g/L) and those in the highest tertile of CRP/albumin ratio, had higher mortality rates, HR 5.35 (95%CI:3.75–7.63) and HR 2.66 (95%CI:212–3.35), compared to participants with high albumin levels (> 36 g/L) and those in lowest tertile of CRP/albumin ratio. These associations were more pronounced in men than in women.

**Conclusion:**

Poor appetite, severe fatigue, low albumin level and a high CRP/albumin ratio were associated with increased mortality rates among patients with advanced cancer. All these variables might be clinically useful for prognostication in palliative cancer care.

**Trial registration:**

Clinicaltrial.gov. Identifier: NCT03038516;31, January 2017.

## Introduction

Loss of appetite is a common symptom in patients with cancer [1, 2]. Cancer-associated cachexia, driven by tumor-induced inflammation, and exacerbated by inadequate nutritional intake, leads to skeletal muscle and fat wasting [3]. Compared to healthy adults, patients with advanced cancer do not respond with physiological counteractive measures to minimize the loss of tissue and weight loss [1, 5]. To identify and treat disease-related eating difficulties, symptoms such as poor appetite, should be prioritized in patients with cancer, since maintenance of body mass index (BMI) is known to decrease mortality [3, 6, 7]. Maintaining body weight is important in all stages of the disease, including the earlier phases [8]. Therefore, nutrition implementations are of utmost importance in clinical practice to possibly extend life expectancy [7–9].

Weight loss is one of the most common signs of nearing death in patients with cancer. This usually occurs the last months and weeks before death. A poor oral intake with increasing difficulties in tolerating solid foods, is one of the symptoms of imminent dying [10, 11]. Besides a changed appetite, the last weeks in life are often characterized by increasing tiredness and fatigue. A loss of appetite commonly correlates with those symptoms in patients with cancer [12]. A first step towards fine-tuning nutrition interventions is to understand the trajectory of appetite, and its association with mortality, in this patient population. Several aspects affect food intake in healthy individuals. One important aspect, that is often overlooked, is the impact of sex hormones, including estrogen, progesterone, and androgens [13, 14]. In addition, previous studies have shown that appetite response may affect men and women differently [15, 16].

To be able to predict remaining survival in patients suffering from advanced cancer is of great importance as it affects clinical decisions. More importantly, prognostication may affect the patient’s decisions and priorities. Although several types of prognostic scores for patients suffering from advanced cancer are available, the accuracy of these varies depending on clinical settings [17, 18]. In clinical practice, a change in albumin levels is often used for assessing prognosis in cancer care, and low levels are associated with a poorer prognosis [19–23]. The Glasgow Prognostic Score includes both C-Reactive Protein (CRP) and albumin levels to predict survival in patients with cancer [24], and these biomarkers have been incorporated in other palliative prognostication tools as well [17]. Another biomarker for prognostication is the CRP and albumin ratio, where a high ratio has been shown to be associated with short-term survival in patients with advanced cancer [25]. In this study, we wanted to assess the association between appetite and mortality and evaluate if appetite can be used as a possible prognostic marker for survival in patients with advanced cancer. To our knowledge, the association between mortality and appetite assessed with the Edmonton Symptom Assessment System (ESAS) has not previously been performed in a palliative care cohort comprising patients in a late stage of their disease. We also set out to evaluate the prognostic value for other markers of cancer progress such as fatigue, change in albumin levels and CRP/albumin ratio. Additionally, we aimed to study possible sex-differences of the associations between these variables and mortality since we recently reported that there were sex-differences in the association between appetite and albumin levels [12].

Thus, the primary aim of this study was to investigate the association between appetite and mortality in patients with advanced cancer. Secondly, we aimed to investigate the associations between fatigue, albumin levels and CRP/albumin ratio and mortality and to study possible sex-differences in these prognostic variables. To this end, post-hoc analyses of previously collected data from the clinical trial Palliative D-study comprising patients admitted to palliative care, were performed.

## Materials and methods

### Study design and population

The present analyses were performed using data from the Palliative-D study comprising 530 patients with cancer admitted to palliative care [26]. The Palliative-D study was a double-blind, randomized placebo-controlled, multi-center trial of vitamin D treatment in palliative cancer care, comprising patients admitted to advanced medical home care (ASIH) in Stockholm, Sweden, during 2017–2020 [26]. Study participants with a vitamin D deficiency were randomized to vitamin D or placebo. However, in the present study all study participants from the screening visit were included, i.e., comprising both patients with vitamin D deficiency and those with sufficient levels that were not randomized. Patients were recruited from three such medical care units in Stockholm: ASIH Stockholm South, ASIH Stockholm North and Stockholm’s Sjukhem. In this care setting patients receive medical care in their own homes by multi-professional teams including nurses, physicians, physiotherapists, dietitians, and social workers. Although there are exceptions, the main criterion for admission is that the patients suffer from a chronic disease in a palliative phase, usually advanced cancer. The median length of care is between three to four months. More details about the unit can be found elsewhere [26, 27].

Patients above the age of 18 years, and diagnosed with advanced cancer, were included. Patients with a life expectancy of < 3 months were excluded from participation. More details about the study design can be found elsewhere [26, 28, 29].

Baseline data on exposures of appetite, fatigue, CRP, and albumin analyzed in the present study were collected from the Palliative-D-study prior to randomization and before any intervention had been performed [29]. The sample size calculation in the Palliative D-study was based on the primary endpoint in the original study, i.e., the effect of vitamin D on pain [30]. No separate power calculation was performed for this post-hoc study. Participants who did not fulfill inclusion criteria, or fulfilled exclusion criteria, were not randomized. The most common reason for not being randomized was that the participant did not have vitamin D deficiency, defined as 25-hydroxyvitamin D levels > 50 nmol/L. Participants who fulfilled the inclusion criteria were randomized 1:1 into an intervention group that received vitamin D 4000 IE/day for 12 weeks or a control group that received placebo for 12 weeks. Participants were followed from date of inclusion of the study, until date of death or end of trial on Sept 30^th^, 2021, whichever came first.

### Patient demographics

Anonymized information on sex, age, date of birth, date of inclusion in the study, cancer type, date of death (when available), randomization arm, and whether the patient was undergoing concurrent cancer treatment, was retrieved from the Palliative D-study eCRF database stored at Karolinska Institutet.

The cancer types of study participants were categorized into four groups based on potential impact on nutritional intake, appetite and eating difficulties: i.e., gastrointestinal, gynecological, head-neck and other cancers (e.g., prostate, lung, and breast cancer). Baseline blood samples were collected for analyses of albumin and CRP levels. Patients filled in the ESAS questionnaire at time of inclusion [31]. Socioeconomic status was assessed by using the average income of the patients’ area of residence as a proxy and divided into two categories: above or below average income. Categorical variables of survival days and study groups were created. Participants were divided into five groups according to survival days: ≤ 30, 31–90, 91–180, 181–365 and ≥ 365 days, respectively. A variable defining belonging to one of three different study groups: not randomized, randomized to study drug, or randomized to placebo, was created depending on if the participant had been randomized or not, and to which study arm they were randomized.

### The edmonton symptom assessment system

The Edmonton Symptom Assessment System (ESAS) questionnaire is a well-established validated instrument used to assess self-reported symptoms in the last 24 h, related to various disease states [31]. It consists of nine core symptoms: pain, fatigue, nausea, depression, anxiety, tiredness, appetite, feeling of wellbeing and shortness of breath [31]. As a tenth question, quality of life (QoL), was included in the Swedish version of the questionnaire [31]. Respondents are asked to assess the symptoms on a scale from 0, meaning less symptoms, (“no suffering”) to 10, more symptoms (“unbearable suffering”). The symptoms for appetite and fatigue were divided into three categories: 0–3 (good appetite/no fatigue), 4–6 (moderate appetite/fatigue) and 7–10 (poor appetite/severe fatigue).

### Biochemical markers

Albumin (g/L) and CRP (mg/L) were analyzed by Karolinska University Laboratory at Karolinska University Hospital, Sweden, using ISO 15189:2012 accredited methods. Albumin levels were categorized into three groups; normal (≥ 36 g/L), moderately decreased (25–35 g/L) and low levels (< 25 g/L). The CRP/albumin ratios were categorized into tertials, as there is no established range for the CRP/albumin ratio.

### Statistical analyses

Descriptive statistics were presented as mean and standard deviation (SD), or number (n) and percentage (%). Independent t-tests were used to assess differences in continuous variables, and chi-squared tests for categorical variables for men and women respectively. Cox proportional hazards model were used to estimate unadjusted, and multivariable adjusted hazard ratios (HR) and 95% confidence intervals (CI:s). Time since inclusion into the study was used as the underlying time scale. Exposures of appetite, fatigue, albumin, and CRP/albumin ratio were analyzed as categorical variables. Adjustments were made for the following confounding factors: sex, age, socio economic status, cancer type, whether the patient was undergoing concurrent oncological treatment or not. To control for potential study specific confounding introduced by the randomization, we also adjusted for study group. Kaplan–Meier plots were used to illustrate the HR function.

In order to evaluate our statistical model we calculated the concordance statistics (c-index) as previously described [32]. This showed a c-index on 0.61 to 0.65, indicating moderate predictive accuracy of the models.

In addition, box-and-whisker plots, as well as a bar graph, were used to present the primary and secondary exposures in relation to the outcome, survival days, along with *p*-values from Mann–Whitney U tests. The CRP/albumin ratio was presented in a bar graph instead of box-and-whisker, as the distribution was skewed.

Statistical analyses were performed using Stata 16.1 (Stata Corporation, College Station, TX, USA), and Graph-Pad Prism version 6.0.

## Results

### Study population

We included 266 men, and 264 women. The mean age of all participants at baseline was 68.6 years. Men were statistically significantly older (mean age 68.6 years) than women (mean age 67.4 years) (*p* = 0.02). The most common cancer types were gastrointestinal (*n* = 225), followed by gynecological (*n* = 39) cancers. A good appetite was reported by 287 (54%), moderate by 146 (28%) and a poor appetite by 97 (18%) patients, respectively. At the end of follow-up, 463 of the 530 participants had died. The median follow-up time was 205 days. Follow up time ranged from 7 to 1420 days (corresponding to 3.8 years). Forty-five study participants (8.5%) had a survival of less than 30 days, 328 (62%) survived 31 to 364 days, and 157 (29.6%) study participants survived longer than 365 days. Participant characteristics are presented in Table 1.

**Table 1:** Characteristics of the study participants (*n* = 530). Appetite and Fatigue was assessed with Edmonton Symptom Assessment System (ESAS), with scores 0–10

	All (*n* = 530) Mean ± SD	Men (*n* = 266) Mean ± SD	Women (*n* = 264) Mean ± SD
Age, years	68.6 (11.1)	68.6 (11.1)	67.4 (11.6)
ESAS Appetite score	3.5 (2.9)	3.4 (2.9)	3.6 (2.9)
ESAS Fatigue score	4.0 (2.7)	4.0 (2.7)	4.0 (2.6)
Albumin, g/L	30.3 (5.2)	30.0 (5.3)	30.4 (5.0)
CRP, mg/L	29.3 (46.4)	32.7 (49.2)	26 (43.1)
CRP/albumin-ratio	1.1 (2.0)	1.3 (2.2)	1.0 (1.9)
	n (%)	n (%)	n (%)
Living area			
Above average income	251 (47.4)	119 (44.7)	132 (50)
Below average income	279 (52.6)	147 (55.3)	132 (50)
ESAS Appetite score			
Good (0–3)	287 (54.2)	149 (56.0)	138 (52.3)
Moderate (4–6)	146 (27.6)	69 (25.9)	77 (29.2)
Poor (7–10)	97 (18.3)	48 (18.0)	49 (18.6)
ESAS Fatigue score			
No/little (0–3)	240 (45.3)	115 (43.2)	125 (47.4)
Moderate (4–6)	183 (34.5)	99 (37.2)	84 (31.8)
Poor (7–10)	107 (20.2)	52 (19.6)	55 (20.8)
Albumin, g/L			
≥ 36	81 (15.2)	52 (19.6)	49 (19.6)
25–35	370 (69.8)	171 (64.3)	177 (67.1)
< 25	79 (14.9)	43 (16.17)	38 (14.4)
CRP/albumin-ratio			
Tertile 1 (0.02–0.17)	181 (34.2)	87 (32.7)	94 (35.6)
Tertile 2 (0.17–0.78)	173 (32.6)	75 (28.2)	98 (37.1)
Tertile 3 (0.78–8.63)	176 (33.2)	194 (39.1)	72 (33.0)
Survival days			
< 30	45 (8.5)	25 (9.4)	20 (7.6)
31–90	103 (19.4)	48 (18.1)	55 (20.8)
91–180	93 (17.6)	51 (19.2)	42 (16.0)
181–365	132 (24.9)	70 (26.3)	62 (23.5)
> 365	157 (29.6)	72 (27.1)	85 (32.2)
Cancer diagnosis			
Gastrointestinal	225 (42)	127 (48)	98 (37)
Gynecological	39 (7.4)		39 (14.8)
Head and neck	11 (2)	9 (3.4)	2 (0.8)
Other	255 (48)	130 (49)	125 (47.3)
Active oncological treatment			
Yes	378 (71.3)	79 (29.7)	73 (27.7)
No	152 (28.7)	187 (70.3)	191 (72.4)
Randomized to vitamin D-study			
Yes	286 (54.0)	146 (55.0)	140 (53.0)
No	123 (23.0)	58 (22.0)	65 (25.0)
Placebo	121 (23.0)	62 (23.3)	59 (22.3)

### Primary exposure: appetite

**Fig. 1:**
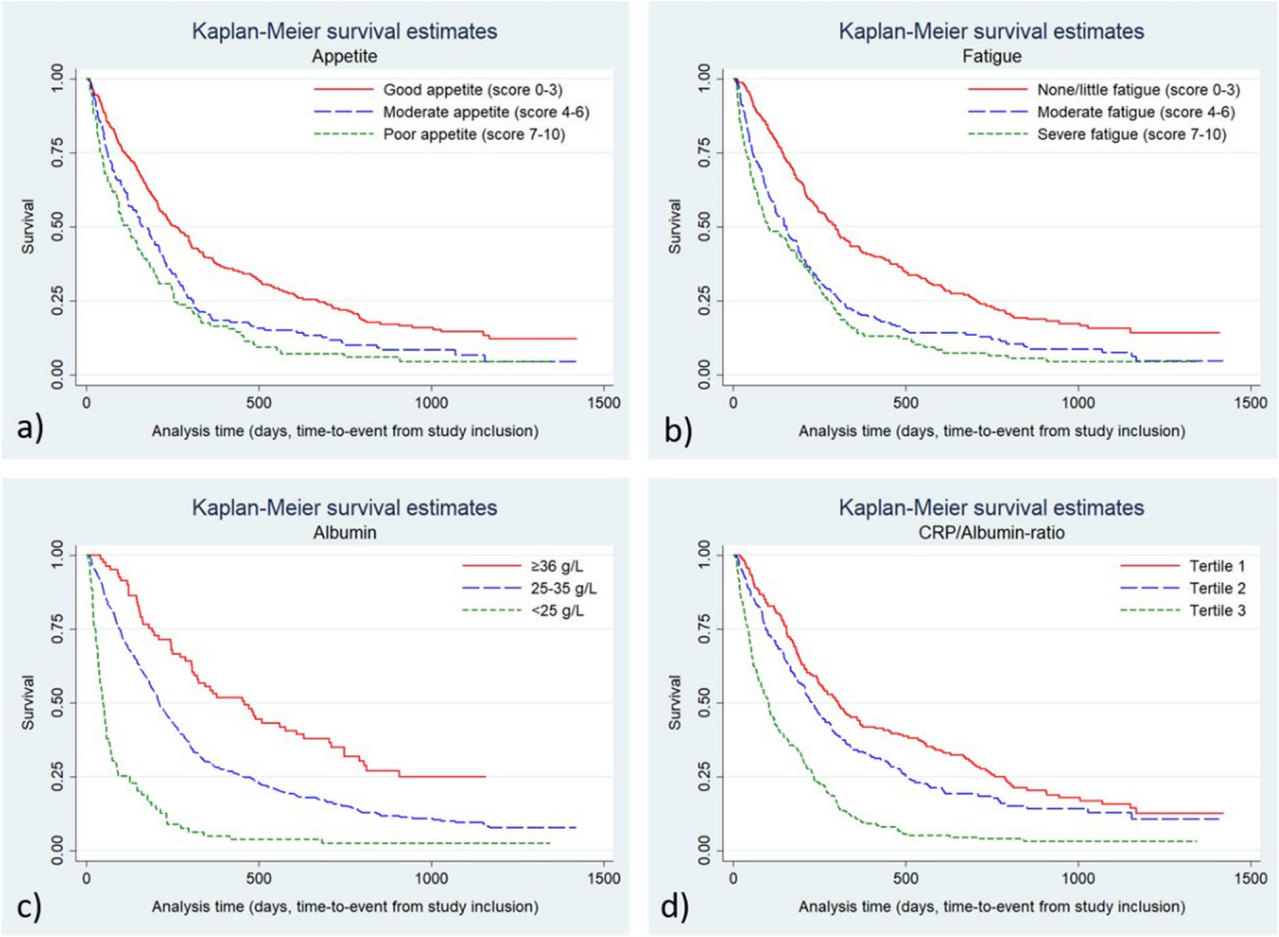
Kaplan–Meier survival graphs for all study participants (*n* = 530) for **a**) appetite, **b**) fatigue, **c**) albumin, and **d**) CRP/albumin-ratio. Appetite and Fatigue was assessed with Edmonton Symptom Assessment System (ESAS), with scores 0–10. The x-axis shows time from inclusion into the study to death or censoring

**Fig. 2:**
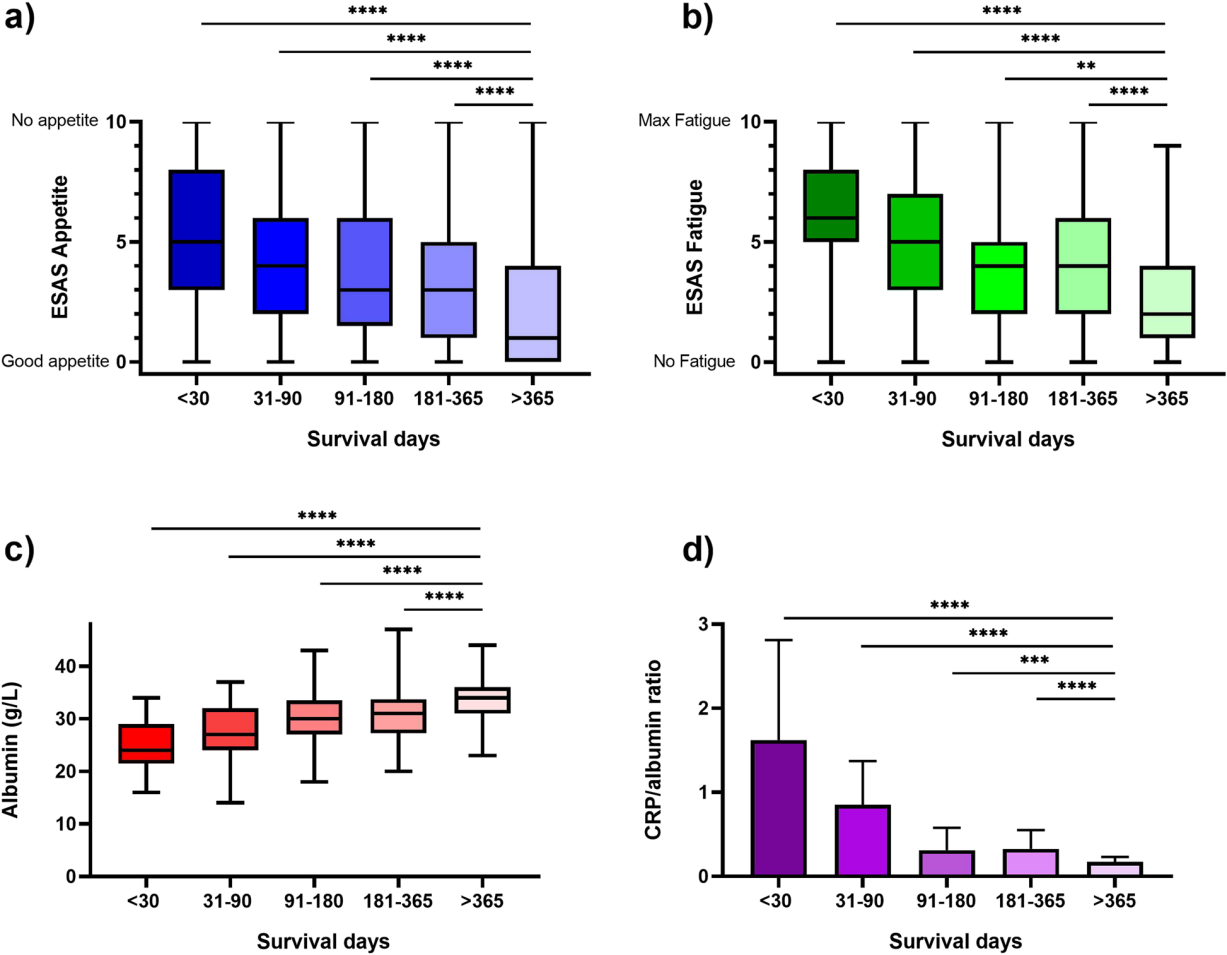
Box and Whisker plots  for all study participants in the Palliativ-D study (*n* = 530) for appetite, fatigue and albumin and a bar chart for CRP/albumin ratio. Appetite and fatigue were assessed with Edmonton Symptom Assessment System (ESAS), with scores 0–10. Statistical analysis was performed using Mann–Whitney test. **** = *p* < 0.0001, *** = *p* < 0.001, ** = *p* < 0.01

Participants who had reported a moderate or a poor appetite had significantly higher mortality rates compared to participants that reported good appetite: adjusted HR 1.44 (95%CI:1.16–1.79) and adjusted HR 1.78 (95%CI:1.39–2.29), respectively (Table 2). Kaplan–Meier graphs of unadjusted mortality rates are presented in Fig. 1a. The Box-and-Whisker’s plot displays the variation of appetite in relation to survival days. Each group was compared with the group that survived more than 365 days from inclusion, i.e. “ > 365 days” (Fig. 2a). The differences between the groups were significant (*p* < 0.0001) suggesting a relation between appetite and survival time.

**Table 2:** Hazard ratios (HR) with 95% confidence intervals (CI) for mortality by categories of appetite, fatigue, and albumin in all participants. Appetite and Fatigue was assessed with Edmonton Symptom Assessment System (ESAS), with scores 0–10

	No of subjects	No of events	Total survival time (person-years)	Crude		Multivariable adjusted^a^	
				HR	(95% CI)	HR	(95% CI)
Appetite score							
Good (0–3)	287	237	111.4	1.00	(reference)	1.00	(reference)
Moderate (4–6)	146	134	38.5	1.54	**(1.24 to 1.90)**	1.44	**(1.16 to 1.79)**
Poor (7–10)	97	92	21.1	1.91	**(1.50 to 2.43)**	1.78	**(1.39 to 2.29)**
Fatigue score							
No/little (0–3)	240	194	100.4	1.00	(reference)	1.00	(reference)
Moderate (4–6)	183	167	47.3	1.72	**(1.40 to 2.12)**	1.69	**(1.36 to 2.08)**
Severe (7–10)	107	102	23.4	2.10	**(1.65 to 2.67)**	1.83	**(1.45 to 2.36)**
Albumin							
≥ = 36	81	58	40.2	1.00	(reference)	1.00	(reference)
26–35	370	328	121.6	1.81	(0.37 to 2.40)	1.89	**(1.42 to 2.51)**
< = 25	79	77	9.7	5.48	**(3.87 to 7.76)**	5.35	**(3.75 to 7.63)**
CRP/albumin							
Tertile 1(0.02–0.17)	181	146	79.9	1.00	(reference)	1.00	(reference)
Tertile 2(0.17–0.78)	173	147	59.7	1.30	**(1.03 to 1.63)**	1.39	**(1.10 to 1.75)**
Tertile 3(0.78–8.63)	176	170	31.4	2.63	**(2.10 to 3.30)**	2.66	**(2.12 to 3.35)**

^a^Adjusted for age, sex, cancer diagnosis, cancer treatment, socioeconomic status, and randomization arm

### Secondary exposures: fatigue, albumin and CRP/albumin Ratio

Participants reporting moderate or severe fatigue had statistically significantly higher mortality rates compared to those reporting little or no fatigue; adjusted HR 1.68 (95% CI:1:36–2.08) and adjusted HR 1.84 (95% CI:1.43–2.36), respectively. Further, we found statistically significant higher mortality rates among participants with moderate to low albumin levels (26–35 g/L) adjusted HR 1.89 (95% CI:1.42–2.51), or low albumin levels (> 25 albumin g/L) adjusted HR 5.35 (95% CI:3.75–7.63), compared to participants with a high albumin level ≥ 36 g/L. We also found that a higher CRP/albumin ratio was statistically significantly associated with higher mortality when comparing tertile 2, adjusted HR 1.39 (95% CI:1.10–1.75), and tertile 3, adjusted HR 2.66 (95% CI:2.12–3.35), with the lowest tertile (Table 2). Kaplan–Meier graphs of unadjusted mortality rates are presented in Fig. 1b, c and d.

The Box-and-Whiskers plots display albumin and fatigue in relation to survival (Fig. 2b, c). The higher the albumin, the less fatigue, and the better survival. The bar graph displays that the lower the ratio CRP/albumin, the longer survival (Fig. 2d).

### Sex-differences in primary and secondary outcomes

**Fig. 3:**
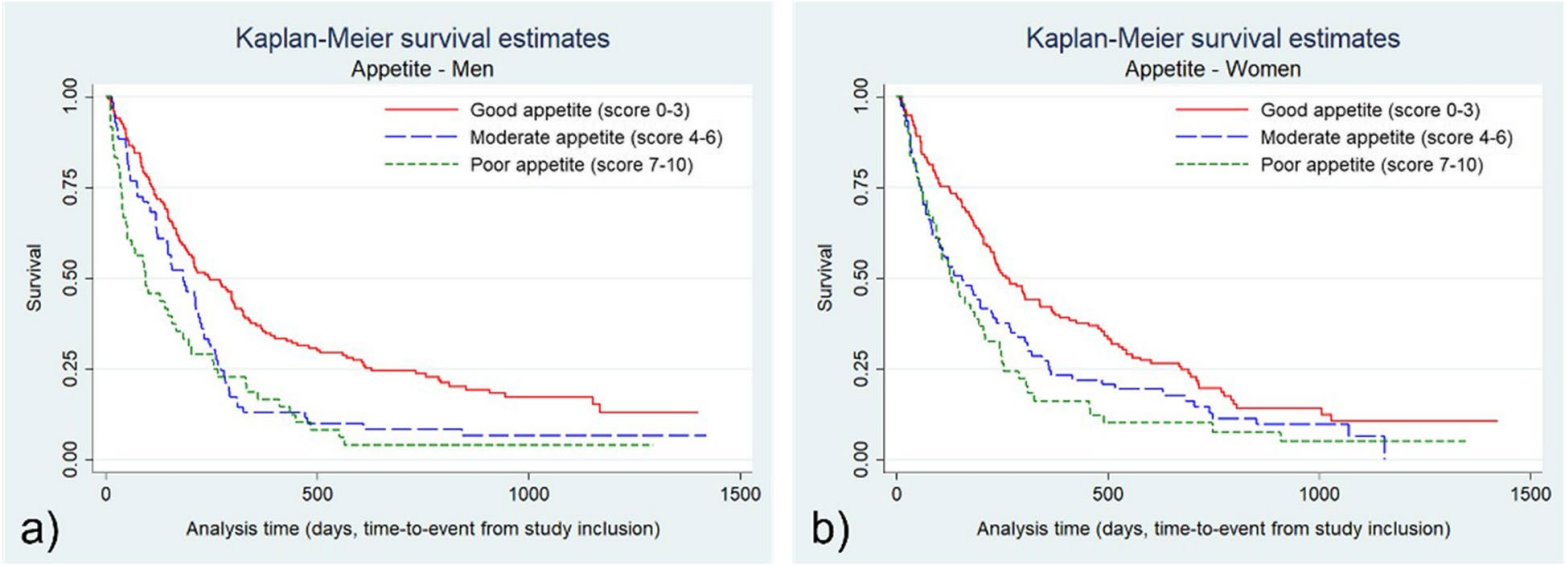
Kaplan–Meier survival graphs for **a**) men (*n* = 266) and **b**) women (*n* = 264) and self-assessed appetite. Appetite and Fatigue was assessed with Edmonton Symptom Assessment System (ESAS), with scores 0–10. The x-axis shows time from inclusion into the study to death or censoring

**Fig. 4:**
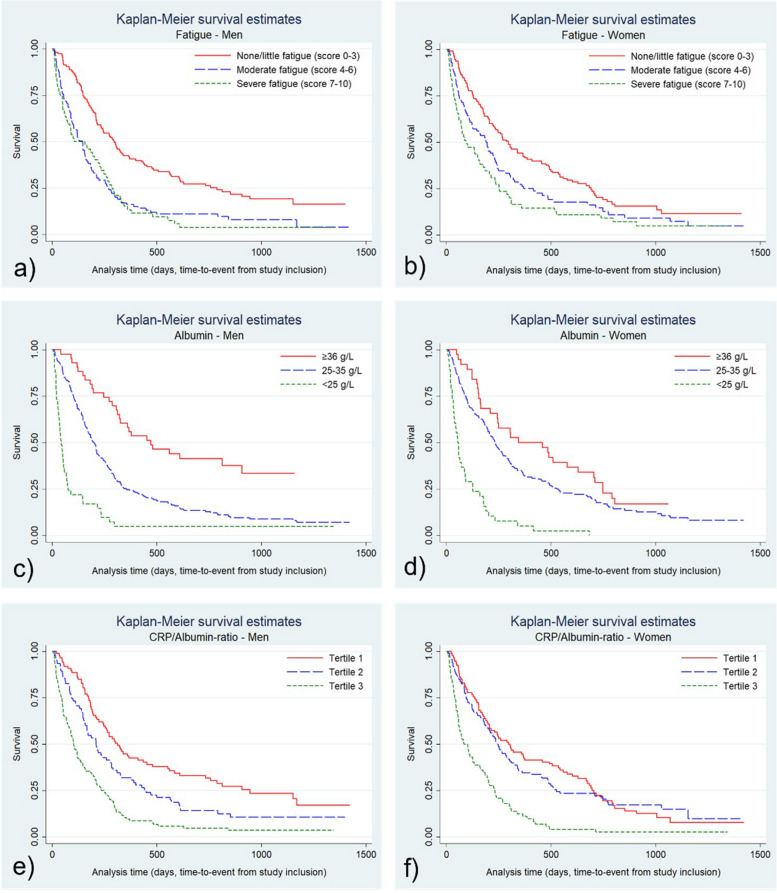
Kaplan–Meier survival graphs for men (*n* = 266) and women (*n* = 264) for **a-b**) fatigue, **c-d**) albumin, and **e**–**f**) CRP/albumin-ratio. Appetite and Fatigue was assessed with Edmonton Symptom Assessment System (ESAS), with scores 0–10. The x-axis shows time from inclusion into the study to death or censoring

Analyses were also performed stratified for sex. We found that a moderately low albumin level (25–36 g/L) was significantly associated with a higher mortality rate in men, adjusted HR 2.46 (95% CI:1.63–3.72), but not in women. Similarly, a moderate CRP/albumin-ratio (tertile 2) was associated with higher mortality in men, adjusted HR 1.74 (95% CI:1.23–2.47), but not in women (Table 3). The Kaplan–Meier graphs for appetite, fatigue, albumin and CRP/albumin ratio in men and women are presented in supplements (Figs. 3 and 4).

**Table 3:** Hazard ratios (HR) with 95% confidence intervals (CI) for mortality by categories of appetite, fatigue, and albumin in men and women separately. Appetite and fatigue was assessed with Edmonton Symptom Assessment System (ESAS), with scores 0–10

	No. of subjects	No. of events	Total survival time (person-years)	Crude		Multivariable adjusted*	
				HR	(95% CI)	HR	(95% CI)
Women							
Appetite score							
0–3 (good)	138	115	52.8	1.00	(reference)	1.00	(reference)
4–6 (moderate)	77	70	21.6	1.44	**(1.07 to 1.93)**	1.41	**(1.04 to1.93)**
7–10 (poor)	49	46	11.8	1.72	**(1.22 to 2.42)**	1.66	**(1.15 to 2.39)**
Fatigue score							
0–3 (no/little)	125	103	49.8	1.00	(reference)	1.00	(reference)
4–6 (moderate)	84	76	24.5	1.46	**(1.08 to 1.96)**	1.63	**(1.20 to 2.21)**
7–10 (severe)	55	52	12.1	1.98	**(1.42 to 2.78)**	1.97	**(1.39 to 2.78)**
Albumin							
≥ = 36	38	31	17.5	1.00	(reference)	1.00	(reference)
26–35	188	162	64.8	1.40	(0.95 to 2.05)	1.43	(0.97 to 2.12)
< = 25	38	38	4.0	4.87	**(2.98 to 7.94)**	4.57	**(2.77 to 7.54)**
CRP/albumin-ratio							
Tertile 1 (0.02–0.17)	94	80	38.6	1.00	(reference)	1.00	(reference)
Tertile 2 (0.17–0.78)	98	81	35.4	1.10	(0.80 to 1.49)	1.14	(0.83 to 1.55)
Tertile 3 (0.78–8.63)	72	70	12.3	2.51	**(1.81 to 3.49)**	2.46	**(1.76 to 3.45)**
Men							
Appetite score							
0–3 (good)	149	122	58.7	1.00	(reference)	1.00	(reference)
4–6 (moderate)	69	64	16.9	1.63	**(1.20 to 2.21)**	1.44	**(1.05 to 1.98)**
7–10 (poor)	48	46	9.3	2.08	**(1.48 to 2.93)**	1.89	**(1.33 to 2.70)**
Fatigue score							
0–3 (no/little)	115	91	50.6	1.00	(reference)	1.00	(reference)
4–6 (moderate)	99	91	22.8	2.02	**(1.50 to 2.71)**	1.84	**(1.35 to 2.51)**
7–10 (severe)	52	50	11.3	2,16	**(1.53 to 3.06)**	1.74	**(1.20 to 2.52)**
Albumin							
≥ = 36	43	27	22.7	1.00	(reference)	1.00	(reference)
26–35	182	166	56.8	2.34	**(1.55 to 3.52)**	2.46	**(1.63 to 3.72)**
< = 25	41	39	5.1	6.44	**(3.91 to 10.61)**	6.80	**(4.07 to 11.35)**
CRP/albumin-ratio							
Tertile 1 (0.02–0.17)	87	66	41.3	1.00	(reference)	1.00	(reference)
Tertile 2 (0.17–0.78)	75	66	24.3	1.55	**(1.10 to 2.18)**	1.74	**(1.23 to 2.47)**
Tertile 3 (0.78–8.63)	104	100	19.1	2.81	**(2.04 to 3.86)**	2.92	**(2.12 to 4.03)**

^a ^age, cancer diagnosis, cancer treatment, socioeconomic status and randomization arm

## Discussion

In this study, we found that patients with advanced cancer reporting a poor or moderate appetite had higher mortality rates than participants that reported a good appetite. Little fatigue, high albumin and a low CRP/albumin ratio were also associated with a lower mortality rate compared to reporting fatigue, and having a high albumin, and a low CRP/albumin ratio. Our results put forward the importance of appetite as a possible prognostic factor of mortality in patients with palliative cancer. In stratified analysis, mortality rates were higher among both women and men with the lowest albumin levels and in the highest tertial of CRP/albumin ratio when compared to those having healthier values of the markers. However, we also found that men, but not women, with moderate albumin levels and CRP/albumin ratio levels, had a higher mortality rate compared to those having healthier values of these biochemical markers. This, tentatively, suggest that these markers might be sex specific.

Loss of appetite is a well-known symptom in patients with cancer, leading to reduced food intake, and weight loss [33]. In concordance with our results, previous studies have shown that reduced food intake, loss of appetite and inflammation measured as elevated CRP-level is a risk factor for over all-survival in patients with cancer [33–35]. However, these previous studies did not differentiate the results by sex.

Our results are also supported by a previous study showing that poor appetite wase associated with mortality six months after hospital discharge in older individuals [36], although that study did not study patients with advanced cancer. Elsewhere it has been reported that old age was significantly associated with more cancer associated symptoms, including loss of appetite [37].

A suggested prognostic score aimed at patients with palliative cancer was introduced and validated in 1999; the Palliative Prognostic Score (PaP score) [38, 39]. The PaP score included anorexia as a variable, as well as other variables such as clinical prediction of survival, Karnofsky Performance Status, dyspnea, total white blood count, and lymphocyte percentage [38, 39]. Thus, this score highlights reduced appetite as an important marker in prognosis. However, to our knowledge, appetite assessed with ESAS and its association to mortality has not been studied before in patients with advanced cancer.

Appetite in advanced cancer might be experienced differently, depending on sex, and therefore, possibly impact the prediction of survival time. In a Canadian retrospective cohort study, female patients with palliative gastric cancer reported less appetite than men. Further, the reported loss of appetite increased as death approached [40]. In a previous study we have demonstrated that cancer-related weight loss was experienced as positive for women but not in men [41]. In contrast, loss of appetite seems to impair QoL in both men and women [12].

A previous study found that an albumin level ≤ 35 g/L was associated with a 3.65 months shorter survival in patients diagnosed with gastric cancer, compared to patients with normal albumin values [42]. This is in line with our results where patients diagnosed with various cancer types, that had an albumin level ≤ 36 g/L, also had a shorter survival time than patients with higher albumin values. This indicates that albumin may predict survival in patients with cancer regardless of cancer type. Our results showed that low albumin levels were associated with high mortality rates in both men and women. However, in analysis of mortality comparing participants with moderately decreased albumin levels of 26–35 g/L to those with a level ≥ 36 g/L, results were only statistically significant among men. In line with this, a previous study conducted in the same cohort, a poor appetite was shown to be associated with a low albumin level in men, but not in women [12]. Thus, the relationship between albumin levels and mortality as well as albumin and appetite seem to be more pronounced in men than in women. However, our results should be interpreted with caution and repeated analysis in larger samples are needed. Nevertheless, the results underline that it may be important to take sex into account when using albumin and CRP/albumin ratio as a prognostic tool. Sex differences in albumin levels and inflammatory markers have been found in other medical conditions too, but, to our knowledge, it has not been studied in patients with cancer [43–45].

Appetite is complex and it often relies on self-reported data. Attempts have been made to identify biomarkers for objective measures, but to date there are no established biomarkers available [46]. Although visual analogue scales have been found to be a reliable and reproducible tool to assess appetite [47], there is no demonstrated link between appetite scores and energy intake [48]. Using self-reported data, such as the ESAS questionnaire, could be a limitation, although ESAS is a validated instrument [31] to collect information about appetite and fatigue. The instrument is used in various health care settings worldwide, as well as for research purposes [31, 49–51]. A limitation in this study is that we did not stratify the data by cancer type to explore appetite by specific cancer types, as the cohort was assessed as too small. However, loss of appetite is a major symptom in advanced cancer, where patients diagnosed with some cancer types, such as cancer in pancreas, are at a heightened risk [52, 53]. Suggested future studies exploring loss of appetite in specific cancer types would contribute valuable knowledge to this field.

A limitation in our study is that only the study participants that had agreed to participate in a randomized, controlled trial on vitamin D treatment were included. Therefore, it is possible that the patients that declined participation contributed to making the cohort less representative. Potentially, patients that were less medically and mentally affected by their disease were more likely to participate. Further, appetite may be affected by vitamin D treatment. Therefore, we adjusted for randomization arm (vitamin D treatment, placebo or not randomized) [54, 55]. However, from our clinical perspective the results are still applicable.

Another limitation to our study is that our data does not contain information about BMI, which has been used as part of a diagnostic criteria for prognostication in patients diagnosed with cancer [7].

A strength of our study is that we included study participants from different socio-economic areas and various demographics without discrimination for age, sex, education, cancer type, ongoing palliative oncological treatment, or performance status, representing patients in general. The range in survival time from a few days to several years shows that patients in both early and late stages of their palliative cancer disease trajectory were included in the study. This strengthens the external validity and may make the results applicable in other palliative cancer care settings as well. However, predicting survival time is a challenge in palliative cancer care, highlighting the significance of finding prognostic markers.

## Conclusion

Loss of appetite is a frequently reported symptom in patients with cancer. It is also an easy measure to collect in clinical practice in patients with advanced disease. The possibility that appetite may be experienced differently in men and women, may require that clinicians adjust the nutrition intervention depending on sex.

Our results indicate that appetite, along with fatigue, and the biochemical markers albumin and CRP/albumin ratio, could be considered valuable prognostic markers that may be used when assessing remaining survival time in patients with cancer. We would also like to stress the importance of taking sex-differences into account when assessing prognostic markers; a field that deserves further studies.

## Data Availability

According to Swedish data legislation access to data can only be made upon request. The request should be addressed to the PI Linda Björkhem-Bergman and will be handled on a case-by-case basis. Any sharing of data will be regulated via data transfer and use agreement with the recipient.
